# Sleep-Disordered Breathing in Children with Recurrent Wheeze/Asthma: A Single Centre Study

**DOI:** 10.3390/children4110097

**Published:** 2017-11-14

**Authors:** Marco Zaffanello, Emma Gasperi, Laura Tenero, Michele Piazza, Angelo Pietrobelli, Luca Sacchetto, Franco Antoniazzi, Giorgio Piacentini

**Affiliations:** 1 Department of Surgery, Dentistry, Paediatrics and Gynaecology, Pediatric Division, University of Verona, Piazzale L.A. Scuro, 10, 37134 Verona, Italy; emma.gasperi@univr.it (E.G.); laura.tenero@univr.it (L.T.); michele.piazza@univr.it (M.P.); angelo.pietrobelli@univr.it (A.P.); franco.antoniazzi@univr.it (F.A.); giorgio.piacentini@univr.it (G.P.); 2 Department of Surgery, Dentistry, Paediatrics and Gynaecology, Otorhinolaryngology Unit, University of Verona, 37134 Verona, Italy; luca.sacchetto@univr.it

**Keywords:** asthma, central breathing control, central sleep apnea, children, in-laboratory overnight respiratory polygraph, obstructive sleep apnea, retrospective study, sleep-disordered breathing

## Abstract

The relationship between asthma and sleep-disordered breathing is bidirectional due to common risk factors that promote airway inflammation. Obstructive sleep-disordered breathing and recurrent wheeze/asthma are conditions that involve the upper and the lower respiratory system, respectively. The aim of the present study was to investigate the sleep disordered breathing in children with recurrent wheeze/asthma. This was a retrospective study concerning children older than 2 years who underwent—between January 2014 and November 2016—an in-laboratory overnight polygraphic study. We match the children between those who do or do not have recurrent wheeze/asthma disease. We examined the clinical records of 137 children. We excluded eight patients because of neurological and genetic conditions. Children with recurrent wheeze/asthma (*N* = 28) were younger (*p* = 0.002) and leaner (*p* = 0.013) compared to non-affected children (*N* = 98). Children with wheeze/asthma and unaffected ones had a similar obstructive apnea-hypopnea index (*p* = 0.733) and oxygen desaturation index (*p* = 0.535). The logistic regression analysis, in which the condition of wheeze/asthma (yes/no) was a dependent variable, while demographic (age, sex, body mass index (BMI) *Z*-score) and polygraphic results during sleep (obstructive apnea-hypopnea index, central apnea index, peripheral oxygen saturation (SpO_2_), and snoring) were covariates, showed that children with wheeze/asthma had higher central apnea index (Exp(B) = 2.212; Wald 6.845; *p* = 0.009). In conclusion, children with recurrent wheeze/asthma showed an increased number of central sleep apneas than unaffected children. This finding may suggest a dysfunction of the breathing control in the central nervous system during sleep. Systemic or central inflammation could be the cause.

## 1. Introduction

Obstructive sleep apnea (OSA) is due to a partial or complete cessation of airflow and oxygen desaturation during sleep [1,2]. The impact on sleep depends partly on the abnormal size of the airway [3]. In children, the major contributor to airway narrowing is hyperplasia of tonsils and adenoids [4] and craniofacial disharmony [5].

The polysomnography confirms the clinical notion of obstructive sleep apnea syndrome (OSAS), also with sleep-disordered breathing (SDB) in asthmatic children [6].

Although children with asthma present a higher risk of SDB, there have still been few sleep studies reporting on this matter [7]. Specifically, asthma is associated with severe OSAS, and, SDB is related to severe asthma. Thus, the relationship between asthma and SDB is bidirectional due to common risk factors that cause airway inflammation [8]. If not attentively controlled, asthmatic children are at a higher risk of severe OSA [9]. In infancy, recurrent wheeze should be differentiated with respect to OSAS [10], and it may also lead to an increased risk of developing future asthma [11]. It is still controversial as to whether or not recurrent wheezing develops asthma if considering also the risk of overestimating or underestimating the symptoms [11]. OSAS and asthma are conditions that affect the upper and lower respiratory system, respectively. So, OSAS and asthma are both characterized by an increased nasal obstruction and vagal tone during sleep, and are detrimental to each other [12]. Recent findings showed that children with OSAS have a significant airway resistance [13], while asthmatic children show a peripheral airway obstruction during symptom-free periods [14]. Lastly, either OSAS or asthma may affect children’s sleep and respiratory system [15].

Central sleep apneas (CSAs) are physiological in newborns and infants: the upper limit is five events per hour. The prevalence of asymptomatic upper CSAs reaches 4.1% in children >1 year of age. Neurological disorders are the main associated conditions [16]. Moreover, an inflammation-mediated activation (e.g., viral or bacterial infection) induces the release of prostaglandins close to brainstem cardiorespiratory-related centers. It depresses the autonomic control networks, including the central drive to breathe during episodes of acute and/or chronic inflammation [17].

The aim of the present study was to investigate the respiratory polysomnographic parameters that characterize SDB in children with recurrent wheeze/asthma and to compare them to the ones of a non-disease condition.

## 2. Materials and Methods

### 2.1. Study Population

A retrospective study, by review of medical records, concerned children older than 2 years with an SDB condition who underwent an in-laboratory, overnight polygraphic study in our department of pediatrics between January 2014 and November 2016.

Reviewed medical records included the in-laboratory overnight polygraphic study and subjects’ age, body growth, asthma, gastroesophageal reflux, as well as genetic and neurological conditions. Children with genetic conditions/syndromes (achondroplasia, etc.) and/or neurological conditions (i.e., Arnold-Chiari malformation) were excluded from the study. Moreover, we performed comparisons between children who had recurrent wheeze/asthma and those who did not. We pooled together wheezing and asthma in children. The primary criteria to enroll children in pooled patients was ≥4 episodes of wheezing in one year [11].

The enrolled children referred were from childhood sleep-disordered breathing, pediatric pneumology ambulatory, or from inpatient settings recovered for wheeze/asthma exacerbation.

The present investigation was based on a preexisting clinical practice, so ethical permission and informed consent were unnecessary. The study was performed following the Declaration of Helsinki and under the terms of relevant local legislation.

### 2.2. Anthropometry

The same trained personnel measured both height and weight using standardized techniques. We calculated body mass index (BMI; weight (kg)/height (m^2^)), BMI percentiles, and BMI *Z*-scores according to age and sex (http://nccd.cdc.gov/dnpabmi/Calculator.aspx).

### 2.3. In-Laboratory Overnight Respiratory Polygraph

The in-laboratory overnight respiratory polygraph study (SOMNOscreen^TM^ PSG, SOMNOmedics GmbH, Randersacker, Germany) monitored nasal airflow, chest and abdominal respiratory movements (thoracic and abdominal belts), arterial oxygen saturation (SaO_2_; digital pulse oximetry), heart rate (HR; finger probe), electrocardiogram (ECG), body position (mercury sensor), and tracheal sounds (microphone). We did not use electroencephalogram (EEG), electrooculogram (EOG) or electromyogram (EMG) techniques. We performed eight channels in the cardiorespiratory assessment in the cases of a respiratory pathology [18]. We applied the device between 6:00 p.m. and 8:00 a.m., and the data were recorded throughout the night. During the test, the children underwent recordings for ≥6 h in a quiet sleep room accompanied by one of their parents [19]. The total sleep time (TST) was calculated from the point at which the child fell asleep to the awakening, and it was reported in a nocturnal diary by the caretaker. No sleep technician was present during the overnight recording. The nursing staff was trained in the assistance. We considered the nocturnal awakenings from the TST computation and removed them from the analysis.

The sleep analysis (DOMINO^®^ software, SOMNOmedics v.2.6.0, Randersacker, Germany) of the entire recording session was manually performed, and some obstructive respiratory events were reported [20]. In particular, the number of obstructive apneas (OA; events/hour) plus hypopneas (H; events/hour) was divided by the hours of TST and expressed as an obstructive apnea-hypopnea index (OAHI; events/hour) [21]. Severe sleep respiratory condition was set at OAHI ≥ 10 events/hour. We calculated the central apnea index (CAI) as the sum of central respiratory events recorded per hour of sleep (events/hour TST), as previously reported [22]. In particular, central apnea (CA) was defined as the absence of nasal airflow and the cessation of respiratory effort, lasting over 20 s or of shorter duration, if associated with a change in plethysmography, as the surrogate of cortical arousals [23], and/or a drop ≥3% oxygen (O_2_) desaturation [16].

We measured the O_2_ desaturations (≥3%; events/hour) from the baseline, mean peripheral oxygen saturation (SpO_2_, %), and smallest SpO_2_ (%). The oxygen desaturation index (ODI; events/hour) is the total number of desaturations divided by the TST. We measured the snoring (% of TST) [15] and the change of position during sleep (events/hour).

### 2.4. Statistical Analysis

We performed the statistical analysis using SPSS Statistics 22.0^®^ software for Windows. The frequency of the distributions (%) was calculated for the categorical variables considered in this study. However, the descriptive statistics (mean, standard deviation (SD)) were calculated for the quantitative variables. Moreover, the chi-square test was used to explore differences in the distribution of the categorical variables between the two groups. Mann–Whitney test was used to explore differences in continuous variables between the two groups. The strength of the association between the two quantitative variables was evaluated by calculating the simple correlation coefficient (Pearson’s test). Statistical significance was set at *p* < 0.05. Binary logistic regression analysis (recurrent wheeze/asthma yes versus no, as the dependent variable) was performed to assess for correlated demographic and respiratory polygraph variables.

## 3. Results

We have examined the clinical records of 134 children, excluding eight patients because of genetic [6] and neurological diseases [2]. None of the children were born prematurely.

Furthermore, we categorized the children (Table 1) according to the coexistence (16/28 had confirmed inhalant allergy) and absence (98) of recurrent wheeze/asthma condition, regardless of whether or not they have had their tonsils and/or adenoids removed (4 versus 18, respectively). These last 22 children performed the polygraph after surgery to investigate for residual SDB [24]. Children with recurrent wheeze/asthma were under treatment with inhaled corticosteroids; no-one took intranasal medications.

A summary of the results of the in-laboratory overnight polygraphic studies of the 126 children is shown in Table 2.

In our sample, children with recurrent wheeze/asthma (*N* = 28) were younger (*p* = 0.002) and leaner (*p* = 0.013) than non-affected children (*N* = 98; Table 1). Recurrent wheeze/asthma children had a comparable OAHI (*p* = 0.733) and ODI (*p* = 0.535), but a higher CAI (*p* < 0.001) than unaffected children (Table 2).

Simple correlation analysis (Pearson’s test) between respiratory polygraph variables was performed to characterize possible correlations (Table 3). In particular, OAHI correlated with ODI in both (*p* < 0.001), whereas OAHI correlated with minimum oxygen saturation (SatO_2_ min) and CAI only in unaffected children (*p* < 0.001). Moreover, ODI correlated with snoring only in the asthma group (*p* = 0.01). Furthermore, ODI correlated with both SatO_2_min and CAI only in the not asthma group (*p* < 0.001).

We did not include two correlated variables (OAHI and ODI) in the computation of regression analysis. The logistic regression analysis in which recurrent wheeze/asthma (categorical variable: 0 = no, 1 = yes) was a dependent variable, and age, sex, BMI *Z*-score, OAHI, CAI, mean SpO_2_, min SpO_2_, snoring (%), and change of position, as covariate, showed that the probability of having a high CAI (Wald 6.845; *p* = 0.009) is increased in the asthma group (Exp(B) = 2.217). The logistic regression analysis in which recurrent wheeze/asthma was a dependent variable and age, sex, BMI *Z*-score, ODI, CAI, mean SpO_2_, min SpO_2_, snoring (%) and change of position as covariate showed that the probability of having a high CAI (Wald 6.614; *p* = 0.010) is increased in the asthma group (Exp(B) = 2.182).

**Figure 1:**
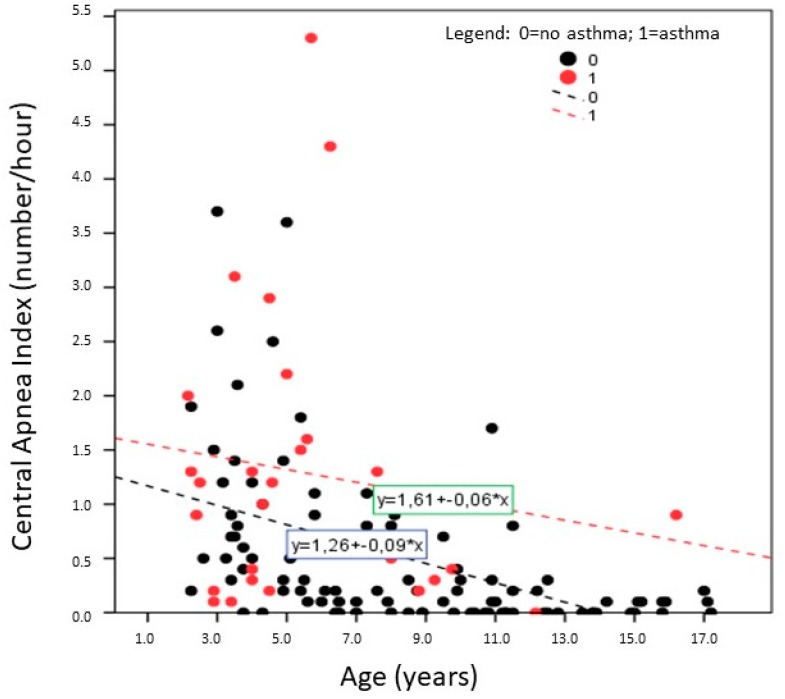
Plotted distribution with regression lines between central apnea index (CAI) and age of the 126 children categorized with recurrent wheeze/asthma (red circle, *N* = 28) or non-asthma (black circle, *N* = 98) condition.

Figure 1 shows the simple linear regression analysis between CAI and age of both children with recurrent wheeze/asthma (red circle) and unaffected ones (black circle). This graphic may suggest that the CAI is higher in children with recurrent wheeze/asthma than in unaffected ones.

In a subgroup of 21 children with recurrent wheeze/asthma and 38 unaffected children, matched for age (4.2 ± 1.4 years versus 4.9 ± 1.6 years, respectively; *p* = not significant (NS)) and OAHI (1.8 ± 1.4 events/hour versus 1.7 ± 1.4 events/hour, respectively; *p* = NS), the test U of Mann–Whitney showed a higher CAI in the former (1.5 ± 1.4 events/hour versus 0.5 ± 0.8 events/hour; *p* = 0.013).

## 4. Discussion

The main findings of the present study were that children with recurrent wheeze/asthma showed an increased number of CSAs compared to unaffected children. Recurrent wheeze/asthma mildly affected the CAI in these children. A sampling of affected and unaffected children, balanced for age and SDB severity, may confirm this observation.

CSA is the absence of chest and abdominal movement, associated with cessation of nasal airflow: the duration of the occurrence is about 20 s or more; if not, it may occur for at least two baseline respiratory cycles and is then associated with an arousal, an awakening, or an oxygen desaturation of at least 3% [20]. The CAI is the number of CSAs per hour of sleep [20]. The authors were supporting the opinion that a CAI > 5 events/hour represents a significant central sleep apnea syndrome (CSAS) [25]. A CAI between 1 event/hour and 5 events/hour is mild CSAS [26]. CSAS can be primary or associated with medical conditions including brainstem pathology [27], heart failure, and prematurity. Therefore, Sholle et al. reported the normative values of CSAs in groups of children with different ages [22]. This method seems to improve sleep pathology identification. Unfortunately, we did not determine central events related to sleep stages because we have not interpreted a proper reading of such events. A full polysomnography (with EEG) could be more helpful in this context in a further prospective protocol design.

Emerging case studies showed that OSAS and CSAs are related. In particular, a CAI ≥ 1 event/hour is more common in children with OSAS, while CSAS (CAI > 5 events/hour) is rare. Moreover, it a significant decrease in the mild CAI after (adeno)tonsillectomy has been observed (preoperative CAI 3.9 ± 2.9 events/hour; postoperative CAI 1.9 ± 4.8 events/hour) [28]. Evidence illustrates an overlap between CSAS and OSAS, including elevated loop gain in patients with OSAS and pharyngeal narrowing during CSAs [29]. Children with asthma occasionally present CSAS [25].

Although the exact cause of CSAS is unknown, this condition likely disrupts the breathing control of the central nervous system [30]. Behavioral factors are largely eliminated during non-REM sleep, leaving the control of breathing to metabolic factors. Partial pressure of carbon dioxide (PaCO_2_) becomes the only stimulus for ventilation during sleep. Respiration-induced changes in the PaCO_2_ play a key role in regulating cerebral blood flow. Alterations in cerebral blood flow triggered by changes in PaCO_2_ are referred to as cerebrovascular reactivity. Children with asthma may experience nocturnal hypoxemia and hypoventilation [31]. In addition, inflammation and remodeling of the airway [32], oxidative stress [33], and systemic inflammation [34,35,36] are reported in asthma patients. Oxidative stress ensues in diverse forms, producing complex structural and functional changes that have implications on cerebral perfusion and permeability of the blood–brain barrier [37,38]. On these bases, we hypothesized that children with asthma may have an increased/additive risk of abnormal central control of the respiration compared to SDB children without recurrent wheeze/asthma.

Nevertheless, the study has several limitations. Inclusion criteria of the polygraph test could affect the results, considering that parents of children with asthma/wheeze may have a tendency to address the problem of sleep disturbance by seeking medical help. We could have a false higher proportion of asthmatics with SDB. In our cohort, 22% are asthmatic, which was higher than expected in this age group, with consequent recruitment bias. In light of this limitation, a prospective study which analyses asthmatic versus non-asthmatic subjects could control this bias. Another limitation of the present study was its retrospective nature. The two selected samples had different ages and SDB severity. Therefore, children beyond two years of age have an increased stability of oxygenation and more stable lung volume [15]. Another limitation of this study is the assessment of the cardiorespiratory analysis during sleep. Sleep stages were not performed, and sleep time was extrapolated according to the caretaker’s statements. Thus, the wakefulness may have been scored as sleep and vice versa [39]. Finally, we pooled together infant wheezing and asthmatic children, even though not all wheeze children would have developed asthma [11].

In conclusion, children with recurrent wheeze/asthma have increased CAI. This finding may suggest a dysfunction of the breathing control in the central nervous system during sleep. We speculate that systemic or central inflammation could be the cause.

## Figures and Tables

**Table 1:** Summary of the demographic and clinical characteristics of the children enrolled in the study. Obesity is defined as a BMI at or above the 95th percentile according to age and sex.

	**Total Patients**	**Recurrent Wheeze/Asthma**	**Not Asthma**	**Chi-Square Test**
	***N*** **(%)**			**(*p*-Value)**
Totals (% males)	126 (56.3%)	28 (46.4%)	98 (59.2%)	0.233
Obesity (*N* (%))	55 (43.7%)	6 (21.4%)	49 (50%)	**0.007**
Allergy (*N* (%))	34 (27.0%)	16 (57.1%)	18 (18.4%)	**<0.001**
GER (*N* (%))	3 (2.4%)	2 (7.1%)	1 (1%)	0.062
**Physical Characteristics**	**Mean ± SD**			**Mann–Whitney Test**
				**(*p*-Value)**
Age (years)	7.8 ± 4.1	5.8 ± 3.4	8.4 ± 4.1	**0.002**
Weight (kg)	39.8 ± 28.3	24.5 ± 14.3	44.2 ± 29.8	**0.001**
Weight-for-Age Percentiles	76.3 ± 29.0	69.6 ± 26.9	78.2 ± 29.5	**0.011**
Weight-for-Age *Z*-score	1.13 ± 1.28	0.70 ± 0.98	1.25 ± 1.33	**0.011**
Height (cm)	127 ± 25	114.1 ± 20.3	131.3 ± 25.2	**0.001**
Height-for-Age Percentiles	65.0 ± 28.2	65.0 ± 29.6	65.0 ± 28.0	0.897
Height-for-Age *Z*-score	0.64 ± 1.2	0.67 ± 1.23	0.64 ± 1.18	0.963
BMI (kg/m^2^)	21.3 ± 7.1	17.6 ± 2.9	22.4 ± 7.6	**0.009**
BMI Percentiles	74.9 ± 29.9	68.0 ± 26.8	76.8 ± 30.7	**0.011**
BMI *Z*-score	1.1 ± 1.2	0.7 ± 1.0	1.2 ± 1.3	**0.013**

BMI: body mass index; GER: gastroesophageal reflux; SD: standard deviation.

**Table 2:** Summary of the in-laboratory overnight polygraphic studies.

Sleep Respiratory Results	Total (*N* = 126)	Recurrent Wheeze/Asthma (*N* = 28)	Not Asthma (*N* = 98)	Mann–Whitney Test
	Mean ± SD			*p*-Value
eTST (h)	8.8 ± 0.9	9.1 ± 1.0	8.7 ±0.9	**0.020**
OAHI (*N*/h)	2.8 ± 5.2	1.7 ± 1.6	3.1 ± 5.8	0.733
CA (*N*/h)	0.7 ± 0.9	1.3 ±1.3	0.5 ± 0.7	**<0.001**
ODI (*N*/h)	2.1 ± 5.7	1.2 ± 1.4	2.3 ± 6.4	0.535
Mean SpO_2_ (%)	97.7 ± 0.9	97.6 ± 1.0	97.7 ± 0.9	0.914
Min SpO_2_ (%)	88.3 ± 8.3	87.4 ± 11.8	88.6 ± 7.1	0.580
Snoring (%)	2.2 ± 5.4	1.0 ± 2.4	2.6 ± 6.0	**0.038**
Change of position (*N*/h)	3.0 ± 1.8	3.4 ± 1.6	2.9 ± 1.9	0.173

TST: total sleep time; OAHI: obstructive apnea-hypopnea index; CA: central apnea; ODI: oxygen desaturation index; SpO_2_: peripheral oxygen saturation.

**Table 3:** Simple correlation analysis (Pearson’s test) of respiratory polygraphic variables.

Respiratory Polygraph Variables	Total (*N* = 126)	Recurrent Wheeze/Asthma (*N* = 28)	No Asthma (*N* = 98)
	*r* (*p*-Value)		
OAHI vs. ODI	**0.807 (<0.001)**	**0.407 (0.032)**	**0.813 (<0.001)**
OAHI vs. SpO_2_ min	**−0.250 (0.005)**	−0.032 (0.873)	**−0.303 (<0.001)**
OAHI vs. snoring	0.038 (0.671)	0.117 (0.552)	0.022 (0.828)
ODI vs. SatO_2_ min	**−0.302 (0.001)**	−0.350 (0.068)	**−0.378 (<0.001)**
ODI vs. Snoring	0.093 (0.298)	**0.480 (0.010)**	0.076 (0.459)

SatO_2_: oxygen saturation.
